# Haptic-Based Neurorehabilitation in Poststroke Patients: A Feasibility Prospective Multicentre Trial for Robotics Hand Rehabilitation

**DOI:** 10.1155/2013/895492

**Published:** 2013-11-12

**Authors:** Andrea Turolla, Omar A. Daud Albasini, Roberto Oboe, Michela Agostini, Paolo Tonin, Stefano Paolucci, Giorgio Sandrini, Annalena Venneri, Lamberto Piron

**Affiliations:** ^1^Laboratory of Kinematics and Robotics, Fondazione IRCCS, Ospedale San Camillo, Via Alberoni, Venezia Lido, 30126 Venice, Italy; ^2^Department of Neuroscience, University of Sheffield and Sheffield Teaching Hospital NHS Foundation Trust, Beech Hill Road, S10 2RX, Sheffield, UK; ^3^AMTC, Universidad de Chile, Avenida Tupper 2007, Edificio AMTC, Santiago, Chile; ^4^Dipartimento di Tecnica e Gestione dei Sistemi Industriali, Università di Padova Stradella S. Nicola, 36100 Vicenza, Italy; ^5^Movement and Brain Laboratory, U.O.F. IRCCS Santa Lucia Foundation, Via Ardeatina, 00142 Rome, Italy; ^6^IRCCS Casimiro Mondino Institute of Neurology Foundation, University of Pavia, Via Mondino, 27100 Pavia, Italy

## Abstract

*Background*. Haptic robots allow the exploitation of known motor learning mechanisms, representing a valuable option for motor treatment after stroke. The aim of this feasibility multicentre study was to test the clinical efficacy of a haptic prototype, for the recovery of hand function after stroke. *Methods*. A prospective pilot clinical trial was planned on 15 consecutive patients enrolled in 3 rehabilitation centre in Italy. All the framework features of the haptic robot (e.g., control loop, external communication, and graphic rendering for virtual reality) were implemented into a real-time MATLAB/Simulink environment, controlling a five-bar linkage able to provide forces up to 20 [N] at the end effector, used for finger and hand rehabilitation therapies. Clinical (i.e., Fugl-Meyer upper extremity scale; nine hold pegboard test) and kinematics (i.e., time; velocity; jerk metric; normalized jerk of standard movements) outcomes were assessed before and after treatment to detect changes in patients' motor performance. Reorganization of cortical activation was detected in one patient by fMRI. *Results and Conclusions*. All patients showed significant improvements in both clinical and kinematic outcomes. Additionally, fMRI results suggest that the proposed approach may promote a better cortical activation in the brain.

## 1. Background

Hand and finger dexterities are fundamental for many activities carried out by a person in order to be independent. Stroke can reduce motor function due to the resulting death of associated brain cells. Stroke leads to permanent neurological impairment in at least 12.6 million people worldwide [1, 2], and in up to 75% of the subjects, motor deficits involve the upper limb [3]. Nowadays, almost all the activities that deal with physical therapy and training tools for rehabilitation have focused on relearning movements of the abilities that the patients had stroke before. Currently, traditional rehabilitative interventions are mainly focused on the passive facilitation of isolated movements or on the promotion of alternative movements to those used before motor diseases [4, 5]. These need to emerge as a consequence of the increasing incidence of stroke patients and the related costs associated to rehabilitation care.

Recent findings from movement neuroscience demonstrated that the human neuromuscular system presents use-dependent plasticity, intended as changes in the pattern of neurons' connectivity [6], not only in healthy but also in neurologically diseased patients, so poststroke patients can experience significant benefits if treated with intensive rehabilitation sessions based on neurophysiologic learning principles [7]. Usually, robotic rehabilitation devices provide passive movement to the patient's arm; however, the use of haptics (i.e., manipulators able to provide force feedbacks), interfaced to VR environments, are becoming a common approach, as they allow intensive, frequent, and repetitive interactive exercise, more coherent with the principles of motor learning. Virtual-reality-based haptic rehabilitation is conceived as the interaction between a haptic device, which consists of a specific manipulator, and a virtual environment [8]. Indeed, a haptic interface enables the patient to move and interact with virtual objects inside a virtual space; hence, a correspondence between the end-effector of the haptic display and a virtual object (avatar) inside the virtual world is verified [9]. This avatar interacts with other virtual objects and the interaction force arising during contacts depends on the “penetration” the avatar performs on the other virtual objects, under a viscoelastic behaviour. As a VR-based application for rehabilitation, static objects are enough for representing the virtual environment [10]. In turn, the interaction force in the virtual environment is reproduced by the haptic device, so the patient receives a force response to his/her motion. This allows the patient to interact with different types of objects, which may have different kinds of properties [11, 12]. Several haptic robots for rehabilitation were developed and tested, such as the MIT-Manus [13, 14], the Mirror Image Movement Enabler (MIME) [15], the ARM-Guide [16], and the BiManu-Track [17].

The design of an effective haptic interface for rehabilitation is often a trial and error procedure and even a small change in the operating scenario or the addition of new features may require large modifications in hardware and software to better respond to the patient's needs. In our prototype the VR-based rehabilitation was conceived as the interaction through a haptic device (i.e., manipulator) with a virtual environment under a viscoelastic behaviour, with interaction forces depending on the “penetration” the avatar performs on the other virtual objects, allowing the patient to perform different motor tasks with different types of objects. Hence, the use of haptic-based therapy highly contributes to regain the mobility that was lost, while for therapists and doctors, this type of computer-based system is an efficient measurement tool, in which the kinematic outcomes for finger and hand rehabilitation (e.g., execution time, trajectory, velocity, and jerk) are accurately evaluated.

In order to merge known benefits of specific stimulation provided by VR and haptics devices, a general framework for virtual-reality- (VR-) based rehabilitation was developed, in which custom designed haptic devices could be easily plugged into the VR rehabilitation environment.

The aim of this study was to evaluate the efficacy of the proposed technological solution for the rehabilitation of hand and fingers motor function in poststroke patients. A feasibility prospective multicentre trial was conducted within a research project supported by the Italian Ministry of Health in three neurorehabilitation hospitals, respectively: Foundation San Camillo Hospital (Venice), Neurological National Institute Foundation “C. Mondino” (Pavia), and Foundation “Santa Lucia” (Rome).

## 2. Material and Methods

### 2.1. Patients

In each hospital, five consecutive stroke patients were enrolled according to the following inclusion criteria: affected by a single ischemic stroke in the region of the middle cerebral artery (MCA) at least six months before the entry; conventional physical therapy treatment received in the early period after stroke; and mild to intermediate motor impairment of the arm (defined as a Box and Blocks Test <45). Clinical history or evidence of memory impairment, neglect, apraxia, or aphasia interfering with verbal comprehension and treated depression were considered as exclusion criteria.

### 2.2. General Framework for VR-Based Haptic Rehabilitation

**Figure 1:**
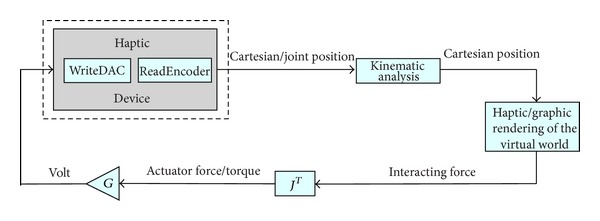
General framework architecture.

The haptic interface consists of a computer (e.g., a PC), a virtual reality engine, a data acquisition board, motor drivers, and a mechanism that constitutes the manipulated part of the haptic device. As shown in Figure 1, the positions (usually joint angles) of the haptic device are acquired via data acquisition board and converted into real world coordinates of the end effector, using a forward kinematic model. Such coordinates are then passed to the VR engine, which is in charge of displaying the exercises on the computer screen and computing the interaction force between the avatar and the virtual objects. The computed force is then converted into force/torque references to the actuators, using the Jacobian matrix of the mechanism, which is obtained by computing the relation between the joint rates and the linear and angular velocity of the end-effector [18].

A general framework is developed in order to achieve the interaction described above. The proposed architecture is implemented inside MATLAB/Simulink. Every block of Figure 1, except the haptic/graphic rendering block, has an S-function associated with it. The VR engine can be selected among many available in the market. For this particular implementation, we chose the Handshake proSENSE Toolbox for haptic and graphic rendering [19]. In a nutshell, the Handshake proSENSE Toolbox adds haptic rendering to standard VRML-based environments. As a MATLAB/Simulink-based product, it works in a drag-and-drop fashion, thus, allowing even untrained users (e.g., doctors and therapists) to quickly develop and test models, and to do on-the-fly modifications on both, virtual environment and exercises, depending on the requirements and performances of the patient. Such general purpose framework achieves two important objectives.

1. Hardware-independent solution: any modification on the hardware implies only minor parameter modifications on the software, mainly the ones related to the Jacobian matrix and the digital and analog channels that are used. This guarantees a fast and easy implementation of any new device, which better fits to some patient's condition and rehabilitation.
2. Online modifications of the elements in the virtual environment: the exercises can be designed in order to fit the requirements and performances of the patient under analysis.

In turn, the proposed framework guarantees a fast and easy implementation for different types of devices, which must handle the corresponding settings and configurations, depending on the type of therapies to be implemented.

Additionally, it provides the haptic and graphic rendering, as well as the control algorithm of the five-bar linkage mechanism, and the external communication with the actuators; these features guarantee a real-time system.

**Figure 2:**
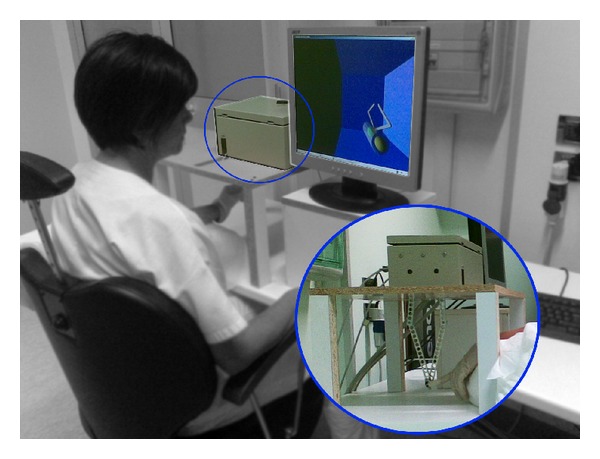
Haptic interface setup. The setup considers a PC with a control algorithm, a VR environment with graphic and haptic rendering, and a five-bar linkage mechanism with finger holder.

The haptic interface implemented in the proposed framework is based on a PC running Microsoft Operating System (Windows XP), with a data acquisition card (Sensoray Model 626 PCI Multifunction I/O Board) that provides the interface to a five-bar linkage haptic device (detailed later), moved by two AC brushless servo motors (Mavilor Motors Model BLS-74) with the relative motor drivers (Infranor CD1-a). The setup and the prototype are shown in Figure 2. The software for controlling the haptic interface was completely developed within MATLAB/Simulink as a real-time application. The external communication to the acquisition board was implemented as an S-function, as well as the kinematic analysis for solving the mechanism, and the calculation of the transpose Jacobian matrix. The visual and haptic feedbacks were implemented using the Handshake proSENSE Toolbox.

**Figure 3:**
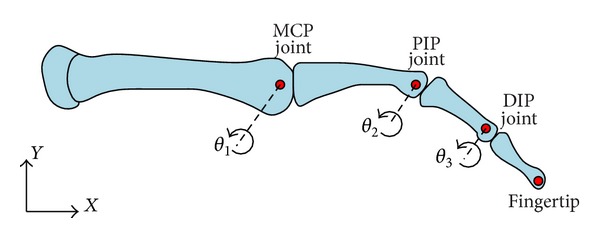
Planar skeletal model of the human finger.

The considerations for the design of the haptic device are focused on the implementation of a single finger haptic display, in which the force is exerted at the fingertip. Herein, all possible movements of each articulation that belongs to the finger are taken into account, along with the reachable workspace of the finger itself. Therefore, this workspace must fit inside the workspace of the haptic device. The design is based on a male index finger (see Figure 3). The average dimensions of the finger were considered and a set of admissible movements (Table 1). This information was derived from the literature and based on statistical data [20].

The chosen mechanism was a five-bar linkage, where one bar is fixed to the frame, and the two cranks, fixed to the frame, are considered the moving members. The proposed mechanism has two active DOF and three passive DOF (rotations) at the fingertip. In this particular case, the driven joints are actuated by AC brushless motors, in which the maximum torque available is 3.4 [N-m], which corresponds to a force of 20 [N] at the fingertip. Each motor has an incremental encoder with a resolution of 32768 pulses per revolution, for which the manipulator position resolution is 0.022 [mm]. The design of the proposed mechanism was based on these three factors.

(i) The haptic device workspace must cover the whole reachable workspace of an average male's index finger.
 (ii) Low inertia.
 (iii) High performance.

The performance analysis is based on the evaluation of the mechanism isotropy (ISO), defined as follows:

(1) $$\begin{array}{c} \mu =\frac{\sigma_{\min}\left(J\left(\Theta \right)\right)}{\sigma_{\max}\left(J\left(\Theta \right)\right)}, \end{array}$$

where *σ*_min⁡_ and *σ*_max⁡_ are the minimum and maximum singular value decomposition values of the Jacobian matrix *J*(Θ) that describes the five-bar linkage mechanism, respectively. Notice that Θ = [*θ*_1_, *θ*_2_, *θ*_3_, *θ*_4_]^*T*^. The mechanism isotropy is a function of the joint angles Θ and the value goes from 0 to 1. An ISO value of 0 means the mechanism is in a singular configuration. A typical singular configuration of the five-bar mechanism is obtained when the links are fully stretched. An ISO value of 1 means maximum performance, which also means that the mechanism can move equally well in all directions.

**Figure 4:**
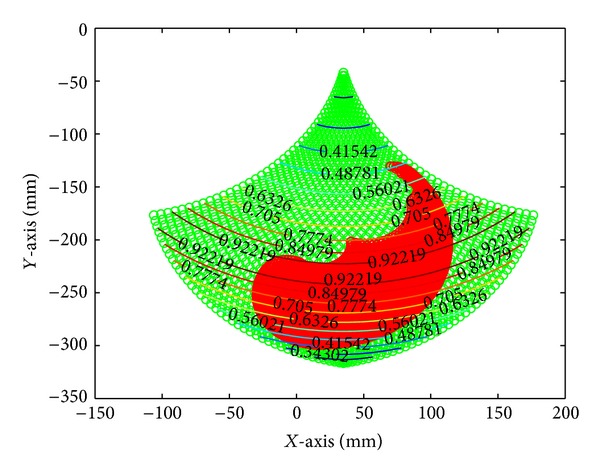
Reachable finger workspace, five-bar linkage workspace and ISO values.

Link lengths were determined by maximizing the mechanism isotropy and by finding the best fitting between the average male's index finger workspace and the haptic device workspace. As a result, under normal conditions, which mean that the finger's motion is without obstacles and/or any load applied on it, the reachable workspace of a single finger is completely covered by the reachable workspace of the mechanism (link length [mm]: *L*_1_ = 140;  *L*_2_ = 180; *L*_3_ = 180; *L*_4_ = 140; *L*_5_ = 70) with ISO values always higher than 0.4 (i.e., good preservation of free movements in all directions) across the whole workspace, as shown in Figure 4.

**Figure 5:**
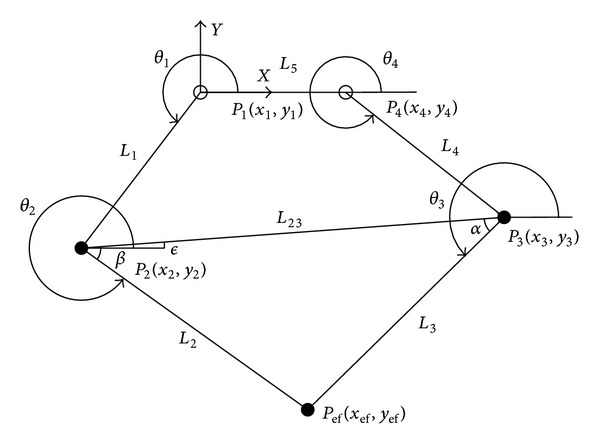
Five-bar linkage mechanism.

In order to determine the end-effector position of the mechanism, forward kinematic analysis has to be done [18]. The angular positions *θ*_1_ and *θ*_4_ are known parameters because they can be read from the encoders that come with the AC brushless motors. The end-effector position of the haptic device is determined with respect to the origin of the *x* − *y* axis, which is placed in *P*_1_(*x*_1_, *y*_1_). Forward kinematics and force analysis are based on Figure 5. The Cartesian position *P*(*x*_ef_, *y*_ef_) of the end-effector of the device is as follows:

(2) $$\begin{array}{c} x_{\text{ef}}=L_{1}\cos\left(\theta_{1}\right)+L_{2}\cos\left(\theta_{2}\right),\\ y_{\text{ef}}=L_{1}\sin\left(\theta_{1}\right)+L_{2}\sin\left(\theta_{2}\right). \end{array}$$

The Jacobian matrix of the mechanism is defined as follows:

(3) $$\begin{array}{c} J\left(\Theta \right)=\left[\begin{array}{cc} a_{11} & a_{12}\\ a_{21} & a_{22} \end{array}\right],\\ a_{11}=-\frac{1}{2}\frac{L_{1}\left(\cos\left(-\theta_{2}+\theta_{1}-\theta_{3}\right)-\cos\left(-\theta_{2}+\theta_{1}+\theta_{3}\right)\right)}{\sin\left(\theta_{2}-\theta_{3}\right)},\\ a_{12}=\frac{1}{2}\frac{L{}_{4}\left(\cos\left(\theta_{2}+\theta_{3}-\theta_{4}\right)-\cos\left(\theta_{2}-\theta_{3}+\theta_{4}\right)\right)}{\sin\left(\theta_{2}-\theta_{3}\right)},\\ a_{21}=\frac{1}{2}\frac{L_{1}\left(\sin\left(-\theta_{2}+\theta_{1}-\theta_{3}\right)+\sin\left(-\theta_{2}+\theta_{1}+\theta_{3}\right)\right)}{\sin\left(\theta_{2}-\theta_{3}\right)},\\ a_{22}=-\frac{1}{2}\frac{L_{4}\left(\sin\left(\theta_{2}-\theta_{3}+\theta_{4}\right)-\sin\left(\theta_{2}+\theta_{3}-\theta_{4}\right)\right)}{\sin\left(\theta_{2}-\theta_{3}\right)}. \end{array}$$

The relationship that links the applied force at the fingertip between the equivalent torques of the electric motors is expressed through the transpose Jacobian of the mechanism:

(4) $$\begin{array}{c} \tau_{F}=J^{T}\left(\Theta \right)F, \end{array}$$

where *F* represents the generalized forces exerted on the end-effector and *τ*_*F*_ represents the torques exerted by the actuators in the joints. Considering the mechanism is used in a vertical plane, it is necessary to determine the equivalent torques in order to compensate the effects of gravity. Therefore, taking into account both, the generated forces at the fingertip and the forces due to the weight, the generated torques transmitted to the motors can be expressed as

(5) $$\begin{array}{c} \tau =J^{T}\left(\Theta \right)F+\eta \left(\Theta \right), \end{array}$$

where *η*(Θ) represents the gravity compensation.

**Figure 6:**
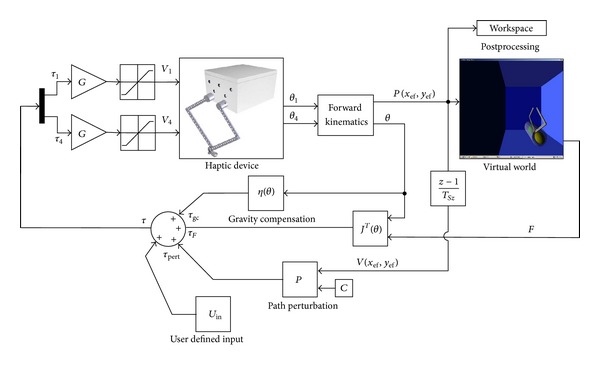
Control diagram of the haptic device and the virtual environment.

An open loop impedance control was used for the proposed five-bar linkage mechanism, in which the input in the physical model of the virtual world is the end-effector position of the device, expressed as a Cartesian position, and the output is the reaction force. The control scheme is described in Figure 6. As the position of the end-effector is known, the physical model algorithm detects a collision with a virtual object. Depending on the properties of the object, the algorithm determines the reaction force. The calculated force is then converted to motor torque through the transpose Jacobian matrix and then the contribution of the gravity compensation is added. As shown in Figure 6, the torque gain *G* is used to convert the signal into volt. A saturation block bounds it, preventing higher exertion forces, and avoiding any possible injury. However, this solution does not always guarantee safety. Hence, a hardware solution is needed in order to achieve safety. Such solution was implemented by putting pressure sensitive safety edges along the moving bars of the mechanism. Using this hardware solution, the safety of the patient is guaranteed when unexpected higher forces occur and/or when the mechanism has unexpected behaviours.

The study was approved independently by the Institutional Review Boards of all participating hospitals and informed written consent was obtained by all participants at the time of enrolment.

### 2.3. Intervention

**Figure 7:**
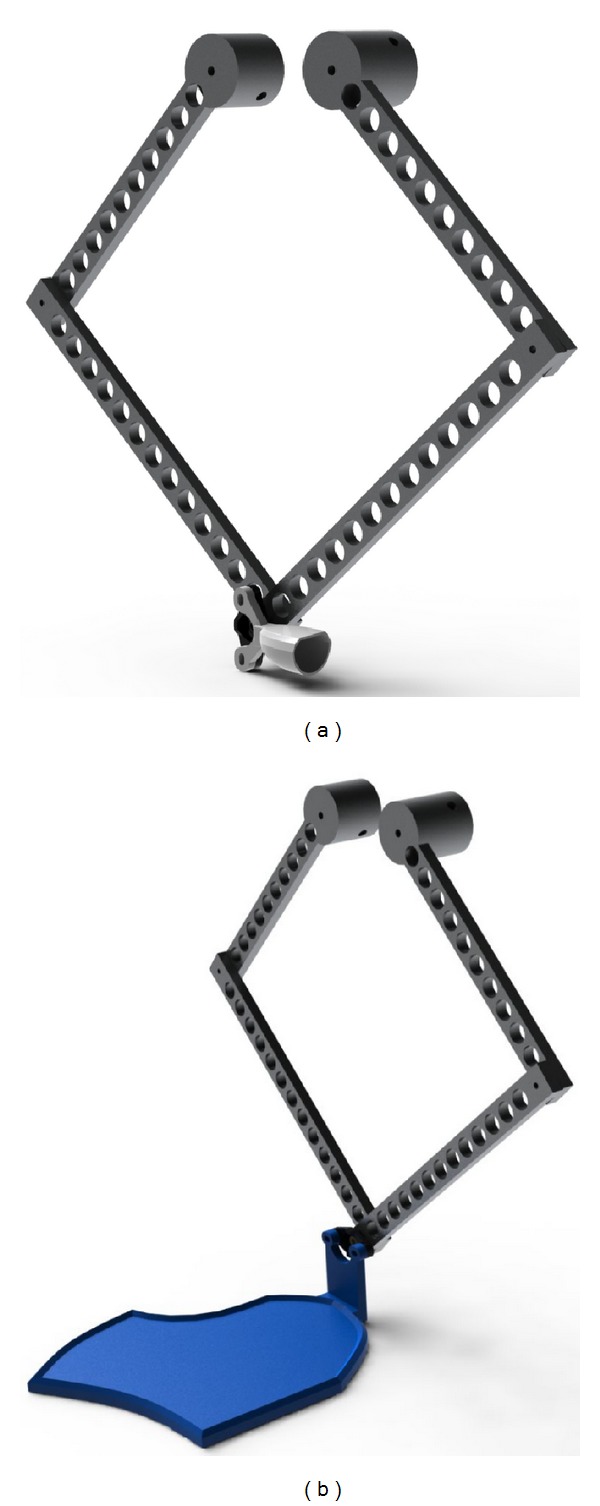
Haptic device for finger/hand rehabilitation. (a) Finger rehabilitation. (b) Hand rehabilitation.

**Figure 8:**
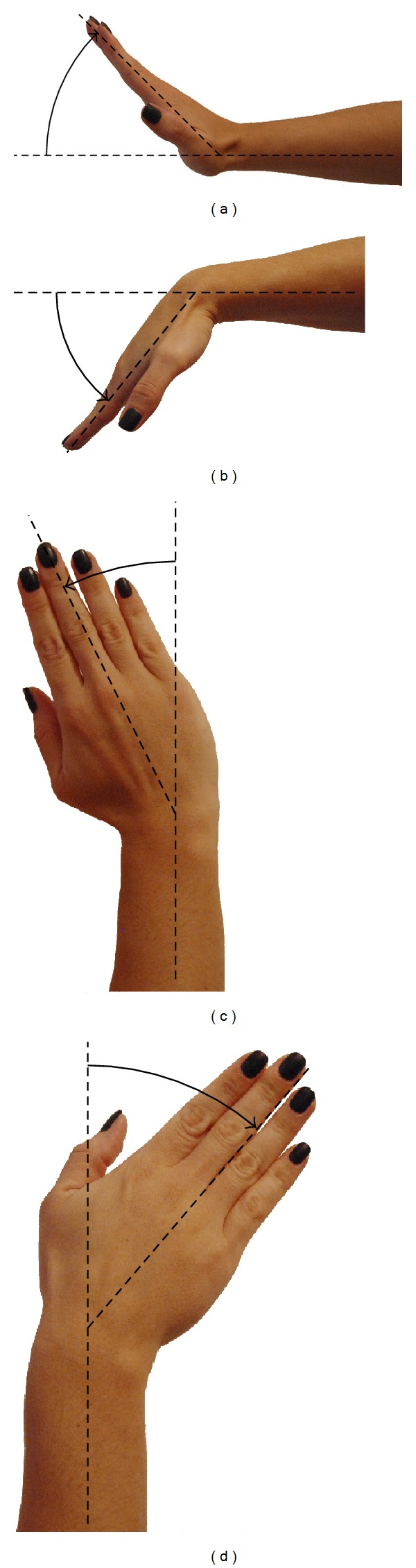
Possible movements for hand rehabilitation. (a) Extension. (b) Flexion. (c) Radial deviation. (d) Ulnar deviation.

**Figure 9:**
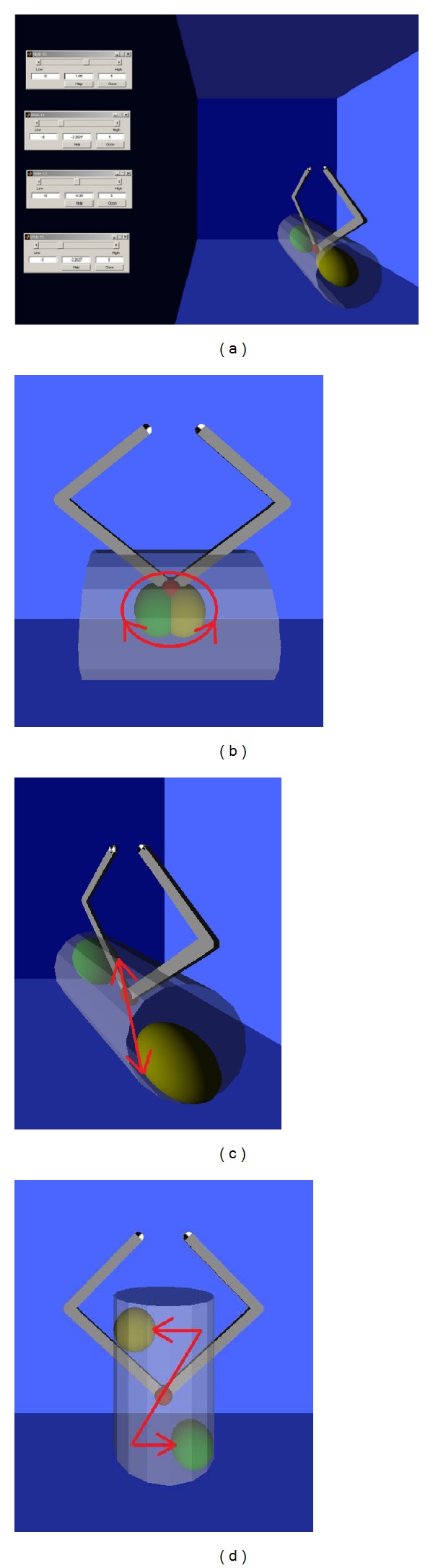
The different scenarios for the exercises. The red arrows indicate the motor task to be accomplished. The environment managed by the therapist for the treatment (a) and examples of exercises proposed in horizontal (b), sagittal (c), and vertical (d) planes.

During the treatment the patients were comfortably seated in an ergonomic chair sustaining the trunk, in front of the haptic device, placed above an ergonomic table. Both, the table and chair, were height adjustable in order to accomplish the best posture for the patients during the therapy (Figure 2). The robot was connected through a spherical passive joint (allowing three rotations around a pivotal point) to a finger or hand holder, depending on the patients' manipulation abilities (Figures 7 and 8). The rehabilitative exercises were developed by the therapist starting from the assessment scenes. The assessment scenes were never proposed as training exercises during the rehabilitation sessions. during the rehabilitation sessions. Following the initial assessment the therapist chose the best configuration of virtual objects to allow the patient to perform the most functional exercise autonomously, according to his/her motor function impairment. The new motor tasks were created by sliding the objects in the virtual environment by means of a graphical interface and adapting it online as the patient progressed during the therapy. During the treatment the subjects were asked to perform several different motor tasks (Figure 9); moreover, in order to interfere with the patient's path during the rehabilitation exercises, artificial perturbations in the system were implemented [21]. The perturbation was tested incrementally asking the patient to counteract the force received while moving on a linear path. Once the patient demonstrated to be unable to control the robot because the safety switches were touched with bars, this value was then set as the maximum threshold reachable. This perturbation was applied orthogonally to the patient's movement direction, affecting their actual path with a force proportional to the end effector velocity exerted by the patient, only in a subset of trials (at least 30% of the overall number of repetitions) and using random forces values within the range settle according to the described procedure. Every treatment session was supervised by the therapist that both instructed the patients on how to accomplish the exercises and also managed online the virtual environment and the user-defined perturbation. The aim of the online robot management by the therapist was to maintain the exercise strain adequate to the real time patients' capability, in order to prevent incoming muscles fatigue and avoiding frustration due to exceeding task challenges.

The treatment protocol lasted 3 weeks, every day 1 session of 45 minutes was provided for 5 days a week. The total amount of sessions performed was 15 for every patient.

### 2.4. Outcome Measures

As the haptics were developed to accomplish exercises targeted to hand and wrist motricity, outcomes describing both the clinical and kinematics domains were chosen. In order to quantify the upper limb motor impairment and hand dexterity of the paretic arm, two scales were administered, respectively: the Fugl-Meyer Upper Extremity (F-M UE) and the Nine Hole Pegboard Test (NHPT). The F-M UE is the most frequently used measure in stroke rehabilitation research. This is an ordinal scale whose scoring ranges from 0 to a maximum of 66 for the upper limb motor performance. The upper limb section has 33 items, which include reflex testing, movement observation, grasp testing, and assessment of coordination. The score for each item is 0 unable to perform; 1 able to perform in part; 2 able to perform [22]. Its clinimetric properties were known and the interrater reliability (*r* = 0.98 to 0.99) and intraclass correlation (ICC = 0.99) were estimated [23]. The NHPT is a test that assesses hand dexterity where the patients, sitting at a table, are asked to take 9 dowels (9 mm diameter, 32 mm long) from the table top and put them into 9 holes (10 mm diameter, 15 mm deep) spaced 50 mm apart on a board. The time to complete this task is recorded; the cut off is set at 50 seconds (when the number of peg placed is recorded). The number of pegs placed per second is then calculated [24]. Also the NHPT clinimetric properties were estimated, respectively, interrater reliability and test retest reliability (IRR and TRT: *r* = 0.68 − 0.99) [23]. 

**Figure 10:**
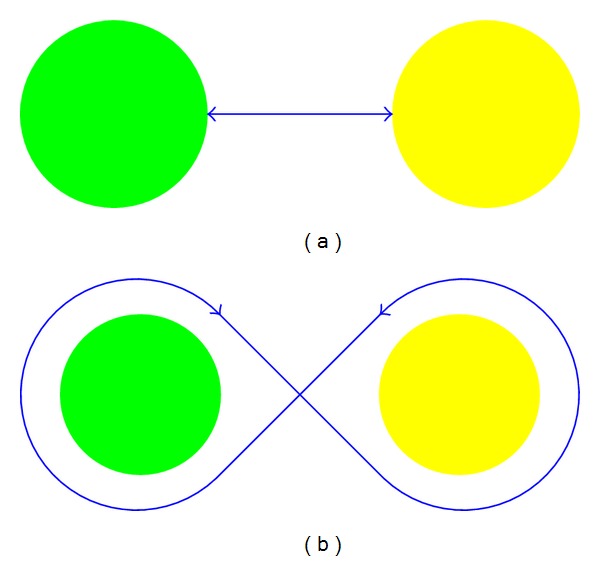
The two motor tasks developed for the kinematic assessment. (a) The task of touching the spheres. (b) The eight shape path.

**Figure 11:**
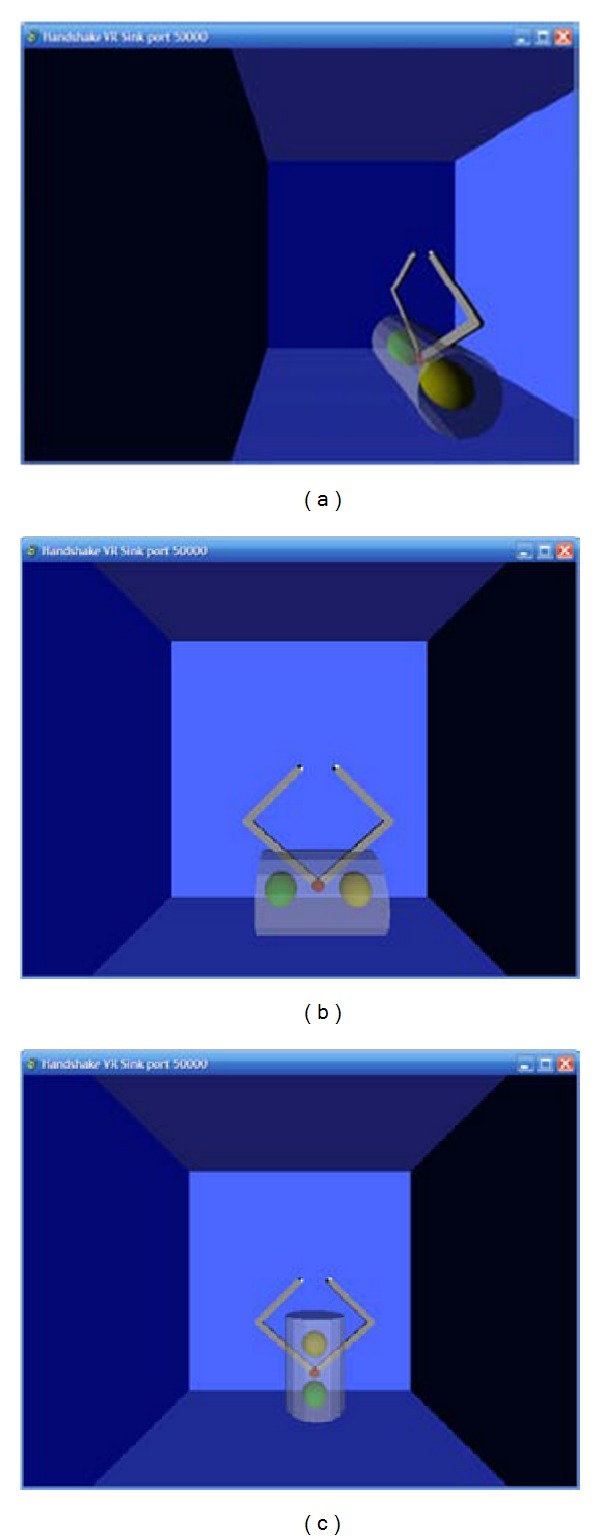
The three different assessment planes. (a) Sagittal. (b) Horizontal. (c) Vertical.

The kinematic assessment of the patients motor behaviour was measured by means of two different motor tasks (Figure 10) each repeated 10 times, both executed in 3 different scenarios (Figure 11), for a total amount of 60 trials.

In the assessment exercises two spheres were displayed in the virtual environment arranged in a planar way, in the 3 different Cartesian planes, and put at the same distance. In the first motor task the patients were asked to touch alternatively the internal surface of the two spheres for 10 times, trying to perform the shorter executable path. In the second motor task the patients were asked to turn around the external surfaces of the two spheres, designing the narrower eight shape paths allowed by the haptics. In order to estimate the motor behaviour changes due to the therapy, we calculated four kinematic parameters describing the motor task performance.

(i) Average time needed to complete every trial [s]: the time the patient takes to execute the task he/she is asked to do.
(ii) Average velocity [m/s] is expressed as the mean of a proportion of the range of motion per unit time of the executed trials.
(iii) Jerk metric [1/s^2^]: this parameter is used in order to characterize the average rate of change of acceleration in a movement [25]. It is defined as follows:
  (6) $$\begin{array}{c} J_{M}=-\frac{1}{V_{\max}}\frac{1}{T}\int \sqrt{{\left(\frac{d^{3}x}{dt}\right)}^{2}+{\left(\frac{d^{3}y}{dt}\right)}^{2}dt}, \end{array}$$
  where *J*_*M*_ is the jerk metric, *T* is the duration, *V*_max⁡_ is the maximum velocity, *d* ^3^ *x*/*dt* is the jerk in *x*-axis, and *d* ^3^ *y*/*dt* is the jerk in *y*-axis.
(iv) Normalized jerk [adim]: this parameter is a unit-free measure, which is used to compare coordination problems in patterns of different durations, shapes and sizes [26].

Consider

(7) $$\begin{array}{c} J_{N}=\sqrt{\frac{1}{2}\frac{T^{5}}{L^{2}}\int {\left(\frac{d^{3}x}{dt}\right)}^{2}+{\left(\frac{d^{3}y}{dt}\right)}^{2}dt}, \end{array}$$

where *J*_*N*_ is the normalized jerk, *T* is the duration, *L* is the length, *d* ^3^ *x*/*dt* is the jerk in *x*-axis, and *d* ^3^ *y*/*dt* is the jerk in *y*-axis.

### 2.5. MRI Acquisition

In order to explore any neurophysiologic effect of the haptics on the cortical reorganization, a functional magnetic resonance imaging (fMRI) study was conducted on one sample patient. Scanning was on a 1.5 Tesla Philips (Achieva) scanner and included acquisition of high-resolution anatomical images, followed by fMRI of two side motor tasks. The patient's hand was fixed to a special cast by means of a binding leaving both the index fingers free to move in flexion-extension directions. The complete paradigm followed a block design in which 30 seconds of flexion-extension of the index finger were followed by 30 seconds of rest. In every run the patient was asked first to fix a black spot for 30 [s] without moving (rest) and then, when the command “move your index finger” appeared, to execute the task continuously in flexion-extension directions for another 30 [s] (movement). These blocks were repeated four times in every run, for a total acquisition of 120 volumes per run. There were 4 runs in which the patient was asked to perform the motor task, alternatively with the right and left index fingers. Scanning parameters included 25 axial 5 [mm] thick slices with no gap, 50 [volumes/series], TR = 2000 [ms], and TE = 40 [ms]. An investigator observed the subject's movements during scanning to verify task compliance. 

### 2.6. Statistical and fMRI Analysis

The descriptive data were reported as mean and standard deviation. The analysis focused both on clinical and kinematics outcomes. For interval scales and ratio measures the variables distribution was explored by means of the Shapiro-Wilk test and parametric (Paired-Samples *T* test) or nonparametric (Wilcoxon signed-rank test) tests were used to determine scores' significant changes after treatment. For ordinal data a nonparametric (Wilcoxon signed-rank test) test was used to determine significant changes in scores after the treatment. SPSS Statistics 17.0 (IBM Inc. Chicago, Illinois) was used for the analysis and statistical significance was considered at *P* = 0.05. Using SPM8 [27], the fMRI images were realigned, normalized to MNI (Montreal Neurological Institute) space, and then spatially smoothed (FWHM = 8 mm). To estimate the cortical activations a general linear model (GLM) was applied (interscan interval: 2 s; microtime resolution: 16; microtime onset: 1) comparing the rest and movement period for the two fingers by means of *t*-contrast (*P* value = 0,01; extent threshold = 20). Finally the results were displayed overlapped on a 3D brain template. 

## 3. Results

The sample was composed of 7 male and 8 female patients with a mean age of 54.00 ± 18.82 years and a mean distance from lesion of 16.59  ±  23.77 months. Six patients were affected by a lesion in the left hemisphere and nine in the right hemisphere. All the patients enrolled were assessed before and after the robotics treatment and completed the treatment protocol. No patients complained of any discomfort due to the interaction with the haptic interface. The baseline and posttreatment values, with their statistical significance, are reported in Tables 2 and 3, respectively, for the clinical and kinematics outcomes. From the explorative analysis both the F-M UE and NHPT were normally distributed (resp., *W* = 0.931, *P* = 0.356 and *W* = 0.969, *P* = 0.887), nevertheless the differences within group for the F-M UE was tested by means of nonparametric statistics, as this scale is considered an ordinal one. In the case of kinematics outcomes all the variables were estimated as normally distributed except the jerk normalized in both exercises (resp., Touch: *W* = 0,814, *P* = 0,007; 8 shape: *W* = 0,652, *P* = 0,000) and the time in the 8 shape exercise (*W* = 0,871, *P* = 0,043). The mean improvement on F-M UE was slightly bigger than 12% (i.e., 8.08 pts.), representing a change close to the minimal clinically important difference of 9 pts., reported for the F-M UE scale [28]. The NHPT increased 19% compared to baseline and despite the significant improvement observed, the lack of reference values in the literature made unreliable speculations on its clinical significance.

For kinematic assessment, both the mean time in trial execution and smoothness, intended as normalized jerk, significantly changed after the treatment, showing an optimization of the trajectories' morphology. We considered the normalized jerk as a smoothness parameter to avoid the differences in patients' functionalities that affect the jerk metric in the easiest task, as displayed in Table 3. On the contrary, the velocity did not change significantly in both the evaluated tasks. This evidence showed that the mean movement velocity should not be a parameter expected to change, in a workspace specifically designed for fingers/wrist movement.

**Figure 12:**
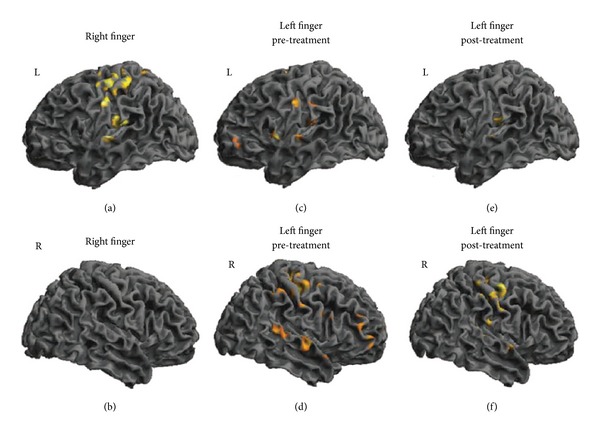
Cortical activation at fMRI. The cortical activation of the healthy (a, b) and impaired (c, d, e, f) finger movements are displayed. The activation of the right finger did not change one month later and was displayed for comparison. Before the treatment, the impaired finger movements induced bilateral activations. After the therapy the ipsilateral activations almost disappeared, while the contralateral activation tuned to M1 as for the healthy finger. L = left hemisphere; R = right hemisphere.

The fMRI analysis (Figure 12) showed that before therapy the requested active finger movement induced, in the affected hemisphere, an activation of the frontal and parietal regions (presumably depending on semantic processes activated together with the motor area) and also an ipsilateral cortical activation of the nonaffected hemisphere. After treatment the ipsilateral activation almost disappeared, while, in the affected hemisphere, all the activations were brought back to a pattern close to the normal one, as for the right finger.

As a result, all patients showed evident improvements in kinematic performance and scored better results in clinical functional-scale assessment, even after a long time after stroke. Additionally, some results shown by fMRI suggest that the proposed approach stimulates the cortical reactivation of the brain.

## 4. Discussion 

The motor therapy provided by means of the haptic interface showed statistical significant improvements in both clinical outcomes. As the F-M UE was known to improve significantly after intensive treatment focused on the upper limb, to our knowledge this is the first time that a significant improvement at NHPT induced by a hand robot therapy was observed in stabilized stroke patients. Similar results were already discussed but in multiple sclerosis patients [29, 30], while significant changing at NHPT in stroke patients were reported for other rehabilitative approaches (e.g., constraint induced movement therapy [31], botulinum toxin [32], passive joint mobilization [33], and task oriented intervention [34]). This result is encouraging and together with the possibility to reproduce reliably the NHPT by haptic interfaces [35], it unfolds new perspectives in assessing quantitatively hand motor recovery after stoke.

For the change in kinematics, we interpreted our result as related to the exercise specificity executed in a hand tailored space. Our setting constrained the patients to a high finalized interaction requiring an optimal end effector control, in this condition others parameters, better describing movement accuracy (such as smoothness), emerged as primary outcome of motor performance. In fact, the velocity in natural motor behaviour should be a movement characteristic mainly in charge of the entire upper limb and strictly dependent from the ability of a person to control trunk stability [36–38]. In our setting, on the contrary, the patients where well ergonomically supported in their posture, minimizing their voluntary control and were asked to enforce voluntary hands' movement to accomplish the exercises.

Finally, the fMRI data supplied a strong neurophysiological contribution to the possibility to induce a fine tuning of the motor cortex, by means of finalized robotics rehabilitation devices. Previous studies [39] already have shown how a finalized motor activity, also robot mediated [40], induced a cortical reorganization, nevertheless in our case it was mandatory to emphasize the continuity between the principles driving the hardware and software haptics' development, the motor rehabilitation protocol applied with the patients, and the experimental paradigm provided for the fMRI study. This data support the evidence that the specificity of the treatment, haptics mediated, could induced a fine tuning of the motor cortex activation, towards a normal pattern. These are the results from a multicenter proof of principle and feasibility study, so more data and controlled studies are needed to confirm robust inferences. The small sample size of the enrolled patients at this time and the absence of a control group should be considered as limitations of the present study. Nevertheless clear and encouraging results emerge from the clinical and neurophysiologic points of view; moreover, the device safety was tested in a clinical setting sustaining its future development.

## 5. Conclusions

A general framework, completely developed in MATLAB/Simulink using the Handshake proSENSE Toolbox, was tested for a five-bar linkage mechanism used as a haptic device, for rehabilitative outcomes. The feasibility and clinical effectiveness of the developed system for a finger/hand rehabilitation program have been demonstrated. The proposed system includes a VR-based interface that allowed to evaluate robustly the finger/hand motion, supporting realtime update parameters and an online storage and retrieval database during the exercise, with the possibility to objectively measure the patients finger/hand functionality, as well as to fairly evaluate the rehabilitation program. The presented haptic interface trains finger and hand motion, while its application grants an excellent motor learning perspective. The presented application can further be improved by extending the haptic interface to more than one finger exercise. This type of application can provide new opportunities for creating more efficient treatments, extending the possibilities of success for the rehabilitation of poststroke patients. The realization of a general-purpose haptic interface, to be used in a multipurpose therapeutic application, is still a largely debated issue, since it is often preferred to use small devices, tailored around a specific pathology. This, however, leads to a large number of devices, each of them characterized by a different mechanical and electronic hardware. This paper addresses the problem of a modular design of the haptic devices for rehabilitation, in which any new device is integrated into the haptic rehabilitation environment as an easily designed S-function in MATLAB/Simulink. In terms of clinical perspectives some methodological issues should be faced in the future, extending the fMRI study both to a large sample of patients undergoing the robot therapy and to multiple followup in time, in order to better understand which plastic cortex mechanism could be really involved; designing bigger clinical trials, in order to avoid a low statistical power; controlling this approach with other recognized effective hands' motor treatments, in order to better estimate the magnitude of effectiveness.

## Figures and Tables

**Table 1:** Finger joint motion ranges.

Finger joint	Angular motion range (degrees)	Link size (mm)
MCP	−90 ≤ θ_1_ ≤ 45	48.3
PIP	−120 ≤ *θ*_2_ ≤ 0	28.2
DIP	−90 ≤ *θ*_3_ ≤ 50	19.1

MCP: metacarpophalangeal; PIP: proximal interphalangeal; DIP: distal interphalangeal.

**Table 2:** Clinical changes due to the treatment.

Functional scales	Before treatment	After treatment	*P* value
Fugl-Meyer upper extremity	45.69 ± 12.88	53.77 ± 8.30	0.002^a^
Nine hole pegboard test	0.21 ± 0.14	0.26 ± 0.03	0.002^b^

The statistical significance value was considered for *P* ≤ 0.05, emphasized in bold.

^a^Wilcoxon signed-rank test.

^b^Paired-Samples *t* test.

**Table 3:** Kinematic changes due to the treatment.

Scenario	Before treatment	After treatment	*P* value
Touch			
Time [s]	4.42 ± 1.62	2.96 ± 0.65	0.002^a^
Velocity [m/s]	0.076 ± 0.021	0.082 ± 0.024	0.074^a^
Jerk metric [1/s^2^]	−11.82 ± 2.56	−12.76 ± 2.44	0.273^a^
Normalized jerk [adim]	1332.55 ± 1336.04	341.00 ± 270.02	0.006^b^
8-shape			
Time [s]	16.64 ± 10.50	10.50 ± 3.96	0.002^b^
Velocity [m/s]	0.100 ± 0.022	0.089 ± 0.017	0.943^a^
Jerk metric [1/s^2^]	−9.50 ± 2.49	−11.64 ± 2.15	0.012^a^
Normalized jerk [adim]	26304.06 ± 41387.15	7851.91 ± 21227.78	0.013^b^

The statistical significance value was considered for *P* ≤ 0.05, emphasized in bold.

^a^Wilcoxon signed-rank test.

^b^Paired-Samples *t* test.

## References

[1] WHO (2011). *World Report on Disability. in Book World Report on Disability*.

[2] Flynn RWV, MacWalter RSM, Doney ASF (2008). The cost of cerebral ischaemia. *Neuropharmacology*.

[3] Prabhakaran S, Zarahn E, Riley C (2008). Inter-individual variability in the capacity for motor recovery after ischemic stroke. *Neurorehabilitation and Neural Repair*.

[4] Van Peppen RPS, Kwakkel G, Wood-Dauphinee S, Hendriks HJM, Van der Wees PJ, Dekker J (2004). The impact of physical therapy on functional outcomes after stroke: What’s the evidence?. *Clinical Rehabilitation*.

[5] Krakauer JW (2006). Motor learning: its relevance to stroke recovery and neurorehabilitation. *Current Opinion in Neurology*.

[6] Sawaki L (2005). Use-dependent plasticity of the human motor cortex in health and disease. *IEEE Engineering in Medicine and Biology Magazine*.

[7] Page SJ, Gater DR, Bach-Y-Rita P (2004). Reconsidering the motor recovery plateau in stroke rehabilitation. *Archives of Physical Medicine and Rehabilitation*.

[8] Broeren J, Dixon M, Stibrant Sunnerhagen K, Rydmark M (2006). Rehabilitation after stroke using virtual reality, haptics (force feedback) and telemedicine. *Studies in health technology and informatics*.

[9] Broeren J, Sunnerhagen KS, Rydmark M (2007). A kinematic analysis of a haptic handheld stylus in a virtual environment: a study in healthy subjects. *Journal of NeuroEngineering and Rehabilitation*.

[10] Broeren J, Bjorkdahl A, Claesson L (2008). Virtual rehabilitation after stroke. *Stud Health Technol Inform*.

[11] Broeren J, Rydmark M, Björkdahl A, Sunnerhagen KS (2007). Assessment and training in a 3-dimensional virtual environment with haptics: a report on 5 cases of motor rehabilitation in the chronic stage after stroke. *Neurorehabilitation and Neural Repair*.

[12] Cameirao MS, Badia SB, Duarte E, Frisoli A, Verschure PF (2012). The combined impact of virtual reality neurorehabilitation and its interfaces on upper extremity functional recovery in patients with chronic stroke. *Stroke*.

[13] Aisen ML, Krebs HI, Hogan N, McDowell F, Volpe BT (1997). The effect of robot-assisted therapy and rehabilitative training on motor recovery following stroke. *Archives of Neurology*.

[14] Fasoli SE, Krebs HI, Stein J, Frontera WR, Hogan N (2003). Effects of robotic therapy on motor impairment and recovery in chronic stroke. *Archives of Physical Medicine and Rehabilitation*.

[15] Lum PS, Burgar CG, Shor PC, Majmundar M, Van der Loos M (2002). Robot-assisted movement training compared with conventional therapy techniques for the rehabilitation of upper-limb motor function after stroke. *Archives of Physical Medicine and Rehabilitation*.

[16] Kahn LE, Zygman ML, Rymer WZ, Reinkensmeyer DJ (2006). Robot-assisted reaching exercise promotes arm movement recovery in chronic hemiparetic stroke: a randomized controlled pilot study. *Journal of NeuroEngineering and Rehabilitation*.

[17] Hesse S, Werner C, Pohl M, Rueckriem S, Mehrholz J, Lingnau ML (2005). Computerized arm training improves the motor control of the severely affected arm after stroke: a single-blinded randomized trial in two centers. *Stroke*.

[18] Spong MW (1989). *Robot Dynamics and Control*.

[20] Venema SC, Hannaford B (2001). A probabilistic representation of human workspace for use in the design of human interface mechanisms. *IEEE/ASME Transactions on Mechatronics*.

[21] Shadmehr R, Mussa-Ivaldi FA (1994). Adaptive representation of dynamics during learning of a motor task. *Journal of Neuroscience*.

[22] Deakin A, Hill H, Pomeroy VM (2003). Rough guide to the Fugl-Meyer assessment upper limb section. *Physiotherapy*.

[23] Croarkin E, Danoff J, Barnes C (2004). Evidence-based rating of upper-extremity motor function tests used for people following a stroke. *Physical Therapy*.

[24] Heller A, Wade DT, Wood VA (1987). Arm function after stroke: measurement and recovery over the first three months. *Journal of Neurology Neurosurgery and Psychiatry*.

[25] Rohrer B, Fasoli S, Krebs HI (2002). Movement smoothness changes during stroke recovery. *Journal of Neuroscience*.

[26] Teulings H-L, Contreras-Vidal JL, Stelmach GE, Adler CH (1997). Parkinsonism reduces coordination of fingers, wrist, and arm in fine motor control. *Experimental Neurology*.

[27] Stephan KE, Weiskopf N, Drysdale PM, Robinson PA, Friston KJ (2007). Comparing hemodynamic models with DCM. *NeuroImage*.

[28] Arya KN, Verma R, Garg RK (2011). Estimating the minimal clinically important difference of an upper extremity recovery measure in subacute stroke patients. *Topics in Stroke Rehabilitation*.

[29] Carpinella I, Cattaneo D, Abuarqub S, Ferrarin M (2009). Robot-based rehabilitation of the upper limbs in multiple sclerosis: feasibility and preliminary results. *Journal of Rehabilitation Medicine*.

[30] Vergaro E, Squeri V, Brichetto G (2010). Adaptive robot training for the treatment of incoordination in Multiple Sclerosis. *Journal of NeuroEngineering and Rehabilitation*.

[31] Brunner IC, Skouen JS, Strand LI (2012). Is modified constraint-induced movement therapy more effective than bimanual training in improving arm motor function in the subacute phase post stroke? A randomized controlled trial. *Clinical Rehabilitation*.

[32] Shaw L, Rodgers H, Price C (2010). BoTULS: a multicentre randomized controlled trial to evaluate the clinical effectiveness and cost-effectiveness of treating upper limb spasticity due to stroke with botulinum toxin type A. *Health Technology Assessment*.

[33] Lindberg P, Schmitz C, Forssberg H, Engardt M, Borg J (2004). Effects of passive-active movement training on upper limb motor function and cortical activation in chronic patients with stroke: a pilot study. *Journal of Rehabilitation Medicine*.

[34] Higgins J, Salbach NM, Wood-Dauphinee S, Richards CL, Côté R, Mayo NE (2006). The effect of a task-oriented intervention on arm function in people with stroke: a randomized controlled trial. *Clinical Rehabilitation*.

[35] Bowler M, Amirabdollahian F, Dautenhahn K Using an embedded reality approach to improve test reliability for NHPT tasks.

[36] Cirstea MC, Levin MF (2000). Compensatory strategies for reaching in stroke. *Brain*.

[37] D’Avella A, Portone A, Fernandez L, Lacquaniti F (2006). Control of fast-reaching movements by muscle synergy combinations. *Journal of Neuroscience*.

[38] Viau A, Feldman AG, McFadyen BJ, Levin MF (2004). Reaching in reality and virtual reality: a comparison of movement kinematics in healthy subjects and in adults with hemiparesis. *Journal of NeuroEngineering and Rehabilitation*.

[39] Nudo RJ, Wise BM, SiFuentes F, Milliken GW (1996). Neural substrates for the effects of rehabilitative training on motor recovery after ischemic infarct. *Science*.

[40] Takahashi CD, Der-Yeghiaian L, Le V, Motiwala RR, Cramer SC (2008). Robot-based hand motor therapy after stroke. *Brain*.

